# Impact of mandatory nucleic acid amplification test (NAAT) testing approval on hospital-onset *Clostridioides difficile* infection (HO-CDI) rates: A diagnostic stewardship intervention

**DOI:** 10.1017/ice.2023.92

**Published:** 2024-01

**Authors:** Winston L. McCormick, Gail Jackson, Sarah B. Andrea, Valerie Whitehead, Tiffany L. Chargualaf, Francine Touzard-Romo

**Affiliations:** 1 Division of Infectious Diseases, Alpert Medical School of Brown University, Providence, Rhode Island; 2 Department of Infection Control, Newport Hospital, Newport, Rhode Island; 3 OHSU-PSU School of Public Health, Portland, Oregan; 4 Lifespan Biostatistics Epidemiology and Research Design Core, Rhode Island Hospital, Rhode Island; 5 Lifespan Clinical Microbiology Laboratory, Providence, Rhode Island; 6 Warren Alpert Medical School of Brown University, Providence, Rhode Island

## Abstract

Misclassification of *Clostridioides difficile* colonization as hospital-onset *C. difficile* infection (HO-CDI) can lead to unnecessary treatment of patients and substantial financial penalties for hospitals. We successfully implemented mandatory *C. difficile* PCR testing approval as a strategy to optimize testing, which was associated with a significant decline in the monthly incidence of HO-CDI rates and lowering of our standardized infection ratio to 0.77 (from 1.03) 18 months after this intervention. Approval request served as an educational opportunity to promote mindful testing and accurate diagnosis of HO-CDI.

*Clostridioides difficile* infection (CDI) remains the most common hospital-acquired infection, causing 30,000 deaths and $4.8 billion in inpatient costs yearly in the United States.^1^ The Centers for Medicaid and Medicare mandate all acute-care hospitals report CDI laboratory-identified events (CDI LabID) via National Healthcare and Safety Network (NHSN). It is a benchmark metric in comparing hospital quality and outcomes. CDI laboratory-identified (LabID) events are considered hospital-acquired and are assigned strictly based on a positive stool specimens collected after 3 calendar days from hospital admission, regardless of symptoms onset or clinical presentation. Sample collection delays commonly result in overdiagnosis of hospital-onset CDI.

Nucleic acid amplification testing (NAAT) is the most widely used diagnostic modality to detect toxigenic *C. difficile* strains. It is highly sensitive but cannot differentiate colonization from infection.^2^ Given the high prevalence of *C. difficile* colonization among hospitalized patients; testing should primarily be considered in patients with at least 3 unformed stools within 24 hours without recent laxative use. Inappropriate orders for testing are common.^1,3,4^ The definition of CDI LabID events and caveats of testing can lead to overreporting and unnecessary clinical and economic impact to patients and payers.

Newport Hospital is a 129-bed hospital that belongs to the Lifespan healthcare system affiliated with The Warren Alpert Medical School of Brown University in Rhode Island. Despite infection control measures, rates of CDI LabID events remained above target. Preauthorization of *C. difficile* testing has been used at other institutions to reduce inappropriate *C. difficile* testing.^1,5,6^ We instituted mandatory *C. difficile* testing approval by infection control to steward appropriate *C. difficile* testing and lower HO-CDI.

## Methods

On July 1, 2019, mandatory approval for CD-NAAT to process any stool sample collected after day 3 of admission to Newport Hospital was implemented. Requests were submitted to infection control via electronic medical record chat message between 8:00 a.m. and 5:00 p.m. or via pager after hours and weekends, in real time. Approval was based on patient-focused discussions regarding testing necessity, considering number of loose stools, duration of symptoms, laxative use, antibiotic use, previous CD testing, and likelihood of other diarrheal causes. There was no hard-stop to order sample testing, but the microbiology laboratory would not process samples until approval was granted. Previously implemented infection control practices and testing initiatives remained unchanged (eg, electronic order sets, laboratory refusal of inappropriate stool consistency, and known positives within 7 days). No additional infection control measures were applied.

We collected demographics, ICU admission, laxatives within 48 hours, antibiotics within 7 days, symptoms of CDI, prior history of CDI and outcomes (hospitalization length, in-hospital mortality of discharge to hospice). We examined distributions of sociodemographic and clinical characteristics within approval status strata by calculating frequencies and percentages of categorical variables and means and standard deviations for continuous variables. We calculated crude relative risk ratios and 95% confidence intervals comparing patients whose test was approved to those whose test was not. Any positive CD-NAAT of a stool sample collected after day 3 of admission was classified as HO-CDI to match CD LabID definition. We performed interrupted time-series analysis (ITS) to assess immediate changes in the trend in CD testing rates and HO-CDI per 1,000 patient days in the 18 months after the intervention (July 2019–December 2020) compared to the 18 months before the intervention (January 2018–June 2019). This analysis was conducted using Stata SE-17 software with the ‘itsa’ module (StataCorp, College Station, TX). We estimated coefficients using ordinary least-squares regression and Newey–West standard errors. Using National Healthcare Safety Network criteria, we calculated preintervention and postintervention standard infection ratios (SIRs). Days of therapy (DOT) per 1,000 patient days for oral vancomycin and fidaxomicin were also calculated.

## Results

In total, 72 stool samples required testing authorization after July 1, 2019, and 65 (90%) were approved. The mean age for testing requests was 67 years; 59% were female; and 72% were admitted outside the ICU. Most (86%) received antibiotics within 7 days of testing request. Baseline demographics, in-hospital mortality, and length of hospitalization were similar in both groups (Table 1). Approved patients were 4 times as likely to have ≥3 loose stools in 24 hours. Although not statistically significant, those who were approved were 28% more likely to have had antibiotics in the past 7 days and 17% less likely to have a prior history of *C. difficile*.

**Table 1.** Baseline Characteristics and Outcomes of Patients Who Required *C. difficile* Nucleic Acid Amplification Testing *(*NAAT) Testing After Day 3 of Hospital Admission Between July 2019 and December 2020

Characteristic	Total (N=72), No. (%)^a^	Not Approved (N=7), No. (%)^a,^ ^b^	Approved (N=65), No. (%)^a^	Unadjusted Relative Risk (95% CI)
Age, mean (SD)	**67.2 (15.2)**	62.5 (24.3)	67.7 (14.3)	1.00 (0.99–1.01)
Total length of stay, mean (SD)	18.8 (17.1)	19.5 (20.5)	18.8 (16.9)	1.00 (0.99–1.00)
Time of test from admission, mean (SD)	9.4 (7.3)	13.8 (12.8)	9.0 (6.6)	0.99 (0.97–1.01)
**Sex**				
Male	29 (41)	3 (50)	26 (40)	Referent
Female	42 (59)	3 (50)	39 (60)	1.03 (0.89–1.20)
**Laxative or stool softener in last 24 h**				
No	65 (90)	6 (86)	59 (91)	Referent
Yes	9 (10)	1 (14)	6 (9)	0.94 (0.69–1.29)
**Intensive care unit**				
No	52 (72)	5 (71)	47 (72)	Referent
Yes	20 (28)	2 (29)	18 (28)	0.99 (0.84–1.18)
**Elevated white blood cell count**				
No	38 (53)	5 (71)	33 (51)	Referent
Yes	34 (47)	2 (29)	32 (49)	1.08 (0.93–1.25)
**Fever in last 24 h**				
No	53 (74)	7 (100)	46 (71)	Referent
Yes	19 (26)	0 (0)	19 (29)	…
**Antibiotics in last 7 d**				
No	11 (15)	3 (43)	8 (12)	Referent
Yes	61 (86)	4 (57)	57 (88)	1.28 (0.89–1.86)
**Diarrhea on admission**				
No	52 (72)	5 (71)	47 (72)	Referent
Yes	20 (28)	2 (29)	18 (28)	0.99 (0.84–1.18)
**≥3 loose stools in 24 h**				
No	8 (11)	6 (86)	2 (3)	Referent
Yes	64 (89)	1 (14)	63 (97)	3.94 (1.17–13.19)
**Prior history of** ***C. difficile***				
No	62 (87)	4 (67)	58 (89)	Referent
Yes	9 (13)	2 (33)	7 (11)	0.83 (0.58–1.19)
**Already tested for** ***C. difficile*** **this admission**				
No	65 (92)	5 (83)	60 (92)	Referent
Yes	6 (8)	1 (17)	5 (8)	0.90 (0.63–1.30)
**In hospital death or discharge to hospice**				
No	62 (87)	6 (100)	56 (86)	Referent
Yes	9 (13)	0 (0)	9 (14)	…

a Units unless otherwise specified.

b Insufficient data for 1 patient in this group.

**Figure 1.**
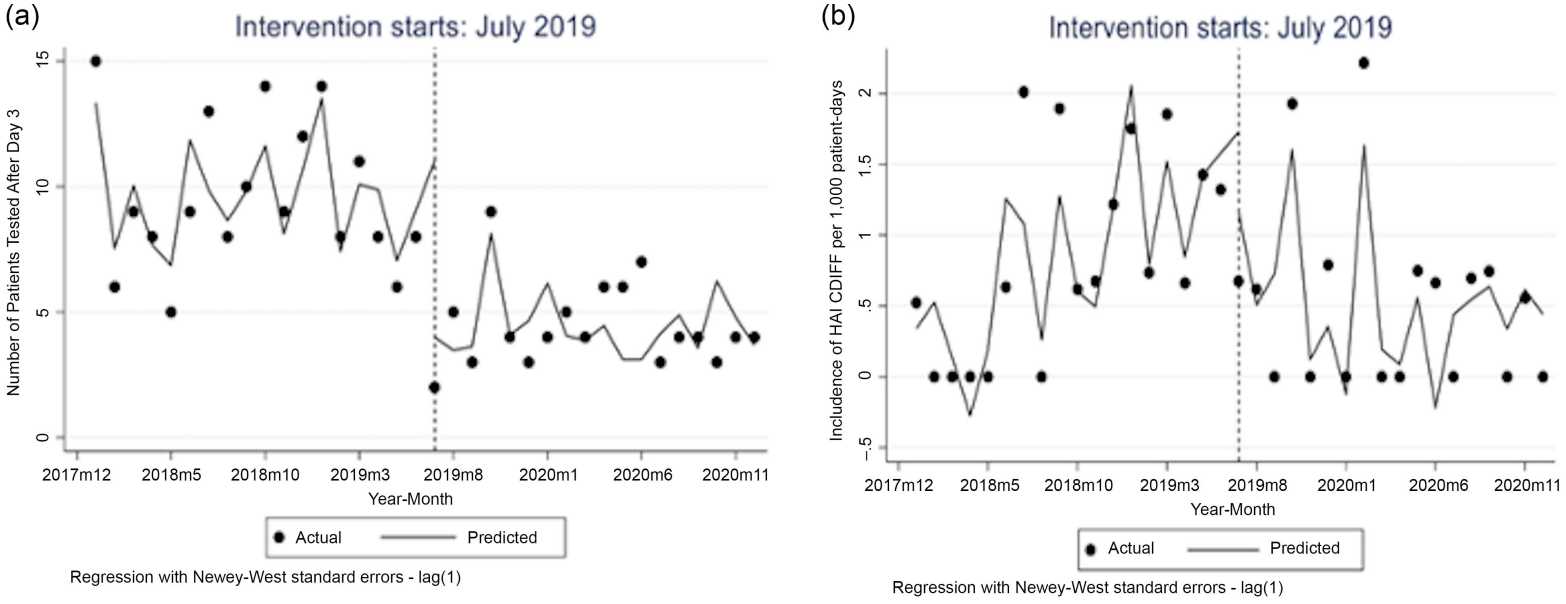
(a) Trend of *C. difficile* (CD) testing after hospital day 3 in the pre-intervention period (January 2018-June 2019) and post-intervention period (July 2019-December 2020). (b) Trend of hospital-onset *C. difficile* infection (HO-CDI) Lab ID event incidence in the pre-intervention period and post-intervention period.

The number of *C. difficile* tests performed after day 3 of hospital admission was 13 per month at baseline with a significant decrease of 6 tests in the first month after intervention (95% CI, −10.0 to −1.35), followed by an insignificant decrease in the monthly trend (−0.14; 95% CI, −0.49 to 0.20) (Fig. 1A). The incidence of HO-CDI before the intervention was 0.51 cases per 1,000 patient days; cases increased monthly prior to July 2019 by 0.11 per 1,000 patient days (95% CI, 0.07–0.16). In the first intervention month, there was a significant decline of 1.16 cases per 1,000 patient days (95% CI, −1.99 to −0.33), followed by a significant decrease in the monthly trend (−0.16 per 1,000 patient days; 95% CI, −0.23 to −0.09). In the preintervention period, there were 23 HO-CDI events versus 13 in the postintervention period. The SIR decreased to 0.77 at the end of the postintervention period from 1.03 in the 18 months prior. DOT per 1,000 patient days for oral vancomycin and fidaxomicin declined from 23.7 before the intervention to 16.2 after the intervention. After the postintervention period, the intervention was discontinued because the hospital adopted 2-step testing.

## Discussion

Approximately 4%–15% of hospitalized patients and 50% of long-term care patients are asymptomatically colonized with *C. difficile*.^1,2,7^ Without 2-step testing to detect toxin production, NAAT must be ordered properly to avoid overdiagnosis of CDI in general. Inconsistent definition and documentation of diarrhea, laxative use dismissal, leukocytosis, and perception of “increased-risk” for CDI are factors that drive inappropriate *C. difficile* testing.^7,8^

Similar strategies have been implemented elsewhere and have been effective in reducing HO-CDI rates.^6,9,10^ Our study resulted in sustained reductions of *C. difficile* testing and HO-CDI rates, allowing us to reach our targeted SIR. Previously, our target had not been achieved despite optimization of infection control practices, implementation of electronic alerts, rejection of stool samples with inadequate consistency, and “soft-stop” orders. CDI treatment DOT did not increase, suggesting that empiric treatments were not started to avoid the approval process.

Most requests were approved, suggesting that providers were mindful about ordering *C. difficile* tests knowing they needed to request authorization. Direct interactions with staff were opportunities to explore alternative causes of diarrhea, confront testing drivers, and avoid duplicate and retesting after recent negative results. For the 7 patients for whom testing was not approved, there was no increased mortality or longer hospitalization, suggesting appropriateness of clinical decision guidance, and repeated testing requests did not occur. Testing denials occurred early in the intervention, indicating that our tool taught testing criteria. Many patients had diarrhea on admission, highlighting opportunities to avoid delay in sample collection that could lead to misclassification of a hospital-acquired infection.

This study had several limitations. It occurred at a community hospital with a small sample size. Such studies may be labor intensive and burdensome at larger institutions. Although small, there is a potential risk of delaying CDI diagnoses given the intervention’s restrictive nature. ITS improves traditional before-and-after study designs by enabling us to decipher changes over time that are beyond secular or seasonal differences, but this design is susceptible to error in the presence of other time-varying factors (eg, compositional changes in patients that present for care).

In conclusion, we successfully instituted a hospitalwide policy in which *C. difficile* NAAT orders to diagnose HO-CDI required approval before processing, encouraging rational use of *C. difficile* testing with associated reductions in HO-CDI cases.
